# An in-silico pan-cancer bulk and single-cell profiling of transcription factors in protein autoubiquitination

**DOI:** 10.1007/s12672-025-03067-0

**Published:** 2025-07-01

**Authors:** Angela Dong, Ayana Rasteh, Panpan Wang, Hengrui Liu

**Affiliations:** 1 Guangdong Provincial Key Laboratory of Traditional Chinese Medicine Informatization, Guangzhou, China; 2 The First Affiliated Hospital of Jinan University, Guangzhou, China; 3 Cancer Research Institute, Jinan University, Guangzhou, China; 4 Tianjin Yinuo Biomedical Co., Ltd, Tianjin, China; 5 Havergal College, Toronto, ON Canada; 6 Archbishop Mitty High School, San Jose, CA USA

**Keywords:** Ubiquitination, Protein, Pan-cancers, Mutation, Prognosis

## Abstract

The protein autoubiquitination has emerged as a significant focus in pan-cancer genetic research due to its potential impact on cancer progression and treatment. Protein autoubiquitination regulates the stability, activity, and localization of involved proteins, playing a crucial role in various cellular processes, including signal transduction, protein quality control, and immune response regulation. This mechanism is vital for maintaining cellular homeostasis and adapting to environmental changes or stress, such as tumor growth. Insights into these processes could lead to novel therapeutic strategies targeting the ubiquitin-proteasome system. This study examines the clinical relevance of transcription factors associated with protein autoubiquitination genes, including CNOT4, MTA1, NFX1, RNF10, RNF112, RNF115, RNF13, RNF141, RNF4, RNF8, TAF1, TRIM13, and UHRF1. Using multi-omics profiling data and Gene Set Cancer Analysis (GSCA) with normalized SEM mRNA expression, the study evaluates differential expression, gene mutations, and drug correlations. The analysis revealed that the single nucleotide variant (SNV) heatmap indicated high mutation frequencies for many of these genes across various cancer types. Gene expression analysis showed limited overall significance, but TAF1 was notably upregulated in uterine corpus endometrial carcinoma (UCEC), while RNF115 and RNF141 were downregulated in the same cancer type. Copy number variation (CNV) profiles exhibited diverse patterns across cancer types, and methylation profiles suggested differences in methylation levels between tumor and normal tissues. Additionally, single-cell transcriptomic analysis uncovered cancer-type-specific functional states. This research highlights the importance of understanding autoubiquitination genes in cancer biology, which may aid in developing effective diagnostic and prognostic strategies. However, the analysis is limited to experimental evidence. However, these findings derive solely from publicly available datasets and lack experimental validation, which may introduce bias. Single-cell analyses cover only a few tumor types, drug-gene relationships remain correlative, and the absence of longitudinal clinical data prevents evaluation of true prognostic value.

## Introduction

According to the International Agency for Research on Cancer (IARC), 1 in 5 people develop cancer during their lifetime and in 2022 alone there were nearly 20 million new cases of cancer [1]. To this day, the prevention of cancer has become one of the most significant health challenges worldwide. Looking at content by cancer types, lung cancer became the most frequently diagnosed cancer globally with almost 2.5 million new cases consisting of 12.4% of diagnoses worldwide in 2022 [1]. It is also highly lethal, accounting for the most number of lives, with approximately 1.8 million deaths (18.7%). Following lung cancer, breast cancer was the second most significant (11.6%) with 6.9% deaths. Other cancers like skin cancer will be diagnosed with 1.5 million new cases in 2022 [1]. Our growing insight into cancer’s intricate biological mechanisms has played a crucial role in the development of more effective cancer therapies. As scientists have unraveled the multifaceted nature of this disease, it has paved the way for significant progress in treatment approaches [2]. Therefore, it is urgent to discover new therapeutic targets and diagnostic/prognostic biomarkers for cancers.

Ubiquitin protein conjugation plays a crucial role in managing both protein turnover and non-proteolytic signaling, impacting a variety of cellular functions such as protein localization, cell cycle regulation, transcription control, DNA repair, and endocytosis [3]. Enzymes involved in ubiquitin metabolism, including E1, E2, E3, E4, deubiquitinases (DUBs), and the proteasome, have been identified as either oncogenes or tumor suppressors in various cancers. Because E3 ligases and DUBs exhibit high substrate specificity, they are of significant interest as potential drug targets in cancer treatment [4]. The autoubiquitination of transcription factors is critical for cancer for several reasons Autoubiquitination can control the abundance of transcription factors in cells. In cancer, this process may be disrupted, leading to abnormal accumulation or rapid degradation of key transcription factors. Ubiquitination can alter the function of transcription factors, potentially enhancing or suppressing their ability to regulate gene expression. This can impact cellular processes like proliferation, survival, and metastasis. Many transcription factors act as oncogenes or tumor suppressors. Their autoubiquitination status can determine whether they promote or inhibit cancer development. Understanding the autoubiquitination of transcription factors can lead to new therapeutic strategies. For example, drugs targeting the ubiquitination machinery or specific transcription factors could potentially treat certain cancers. Some transcription factors involved in cell cycle regulation are controlled by autoubiquitination. Disruption of this process can lead to uncontrolled cell division, a hallmark of cancer. Last but not least, autoubiquitination of certain transcription factors is involved in cellular stress responses. Cancer cells often manipulate these pathways to survive in harsh tumor microenvironments.

A total of 14 transcription factors genes associated with autoubiquitination were collected from the Gene Ontology database, including CNOT4 [5], MTA1, NFX1 [6], RNF10 [7], RNF112 [8], RNF115 [9], RNF13 [10], RNF141, RNF4, RNF8, TAF1, TRIM13, and UHRF1. For instance, TAF1, a subunit of the general transcription factor TFIID, plays a role in chromatin remodeling, specifically in regulating gene transcription [11]. It does this by recognizing acetylated lysines on histones, which are proteins that package DNA into chromatin, according to a research article published in the journal “Nucleic Acids ResearchUnderstanding these genes associated with protein autoubiquitination in cancer is crucial for comprehending ubiquitination-related tumour development and exploring it as a potential therapeutic avenue for future cancer treatments. Analyzing multi-omic profiling data, we extensively studied protein autoubiquitination involving these 14 genes across over 9000 samples from 33 cancer types. Our lab has previously applied similar analysis to understand the biomarker potential of different gene sets [12–16]. This study aims to comprehensively analyze this gene set across different types of cancer and their clinical associations, laying the groundwork for future therapeutic strategies. We will discover new therapeutic strategies for cancer treatments by testing these genetic relationships.

## Methods

### Data acquisitions

This study obtained its genetic data from the Gene Set Cancer Analysis (GSCA ), which is an integrated platform for genomic, pharmacogenomic, and immunogenomic gene set cancer analysis. The data for the RSEM normalized mRNA expression data, survival data, methylation data, copy nucleotide variation (CNV), and single nucleotide variation (SNV) data were gathered from The Cancer Genome Atlas (TCGA) database [17]. The reverse phase protein array (RPPA) [18] was collected from the cancer proteome atlas (TCPA) [19].

### Survival analysis

SNV, methylation, & mRNA expression and survival data were merged via sample barcode, and tumour samples were categorized as being mutant when a specific gene was mutated in those samples. CNV data from 11,495 samples were processed through GISTIC2.0 [20]. R package survival and GSCA were used to establish survival time and status, and the Cox proportional-hazards model & Log-rank tests were used to determine survival differences between groups. For methylation survival analysis, a Logrank test was performed to determine if the survival difference was significant.

### Expression analysis

CNV and mRNA data were integrated using the TCGA barcode; Spearman correlation analysis was employed to assess the relationship between mRNA expression and CNV. Methylation levels were similarly evaluated using Spearman correlation analysis. Differential analysis of methylation and mRNA expression between tumour and normal cells was conducted using the t-test. Subtype analysis was performed using the Wilcoxon test and ANOVA.

### Pathway analysis

The TCPA database’s Reverse Phase Protein Array (RPPA) was utilized to assess pathway activity in 7,876 cancer-related samples. Proteins were denatured with SDS and arrayed on nitrocellulose-coated slides using specific antibodies. Ten established cancer pathways—Estrogen Receptor (ER), Androgen Receptor (AR), PI3K/AKT, RAS/MAPK, Epithelial-Mesenchymal Transition (EMT), TSC/mTOR, Receptor Tyrosine Kinase (RTK), Cell Cycle, and Apoptosis—were evaluated by calculating pathway activity scores (PAS). The student T-test was employed to analyze PAST differences between groups based on median gene expression levels. Significance was determined using False Discovery Rate (FDR) adjusted P-values (FDR ≤ 0.05). A gene was considered to activate a pathway if PAS (High expression group) exceeded PAS (Low expression group); otherwise, it was deemed inhibitory.

### Single cell analysis

Single-cell functional state analysis was performed using the CancerSEA web-based platform (http://biocc.hrbmu.edu.cn/CancerSEA/), which integrates single-cell RNA-seq data from various tumor types to assess correlations between gene expression and 14 predefined cancer-related functional states. We selected the default dataset provided by CancerSEA and retrieved Spearman correlation values for each cancer type and functional state pair. Correlation coefficients and associated P-values were visualized using a bubble plot, where circle size represents the strength of the correlation and color denotes the direction (red for positive, blue for negative). Only statistically significant correlations (*P* < 0.05) were considered for interpretation.

### Immune association analysis

The immune cell’s infiltrates and gene set expression levels are estimated by the Immune infiltration and GSVA score. The Immune infiltration scores are evaluated through 24 immune cells and ImmuCellAI [21]. In contrast, the GSVA score represents integration levels of expression, where it could indicate that the overall gene set in the tumour is higher or lower. The Spearman correlation value between the infiltrating immune cell and the entered gene set’s GSVA score was applied.

### Drug sensitivity analysis

The protein autoubiquitination gene set’s correlation with drug sensitivity was assessed using a cut-off of remarkable significance (*p* < 1e-5). Data on drug sensitivity and mRNA expression were combined. To find the link between medication IC50 and gene mRNA expression. Additionally, Genomics of Drug Sensitivity in Cancer (GDSC) matching mRNA gene expression was employed.

### Statistical analysis

R software version 4.0.3 was used for all statistical studies. The Spearman correlation test was used for correlation analysis, and the Cox proportional hazards model was used to determine the hazard ratio (HR) and survival risk. For two sets of data, unless otherwise indicated, the rank-sum test was used, and a P-value of less than 0.05 was regarded as statistical.

## Results

### Single nucleotide variation analysis

**Fig. 1:**
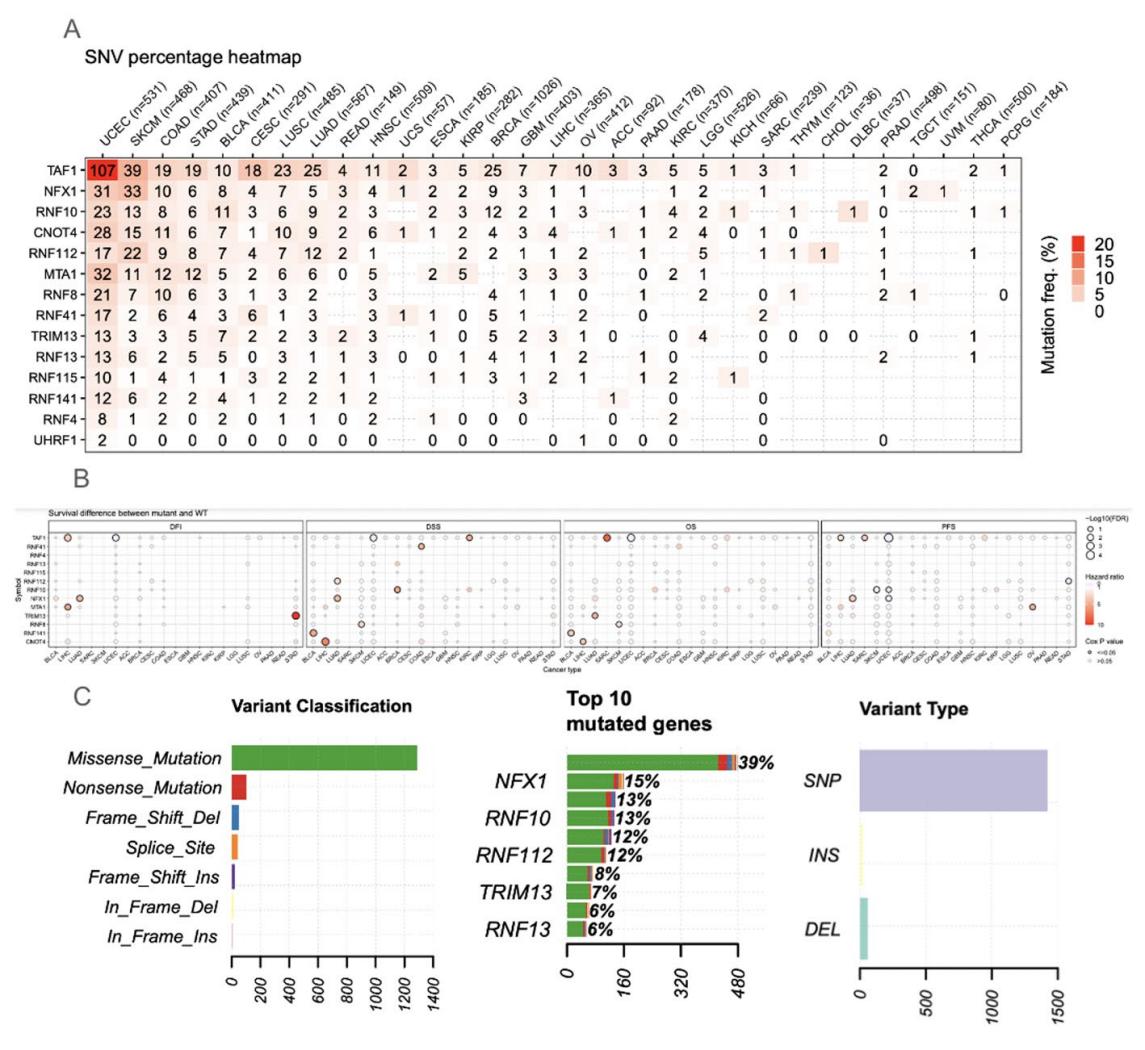
Mutation landscape and prognostic impact of autoubiquitination-associated genes in pan-cancer. A: Heatmap illustrating mutation frequencies. Each number denotes the presence of mutations in a specific cancer sample; 0 indicates no mutations in that genomic region. Colours indicate varying mutation frequencies across cancers. B: Survival plot comparing single nucleotide variants (SNVs) between mutated and normal genes within the protein autoubiquitination-associated gene set. C: Summary of SNV classes and mutation types for protein autoubiquitination in cancer

The single nucleotide variation (SNV) profiles of the protein autoubiquitination gene set were analyzed. The SNV heatmap showed that mainly TAF1 had a high SNV mutation frequency across some cancer types. The other majority had slight SNV mutation frequencies. Particularly, the genes that showed the highest mutation frequency across all cancers overall were TAF1, NFX1, and RNF10. Additionally, TAF1 was mutated in over 20% of UCEC samples. The cancer types that were most impacted were UCEC, SKCM, and COAD (Fig. 1A). According to the SNV landscape, the most common SNV mutations were missense mutations, with nonsense mutations being the second leading type (Fig. 1C). The profile of the survival associations with the SNV of these genes showed that there may be a relation between TRM13 and STAD as it relates to the survival times of patients (Fig. 1B). Additionally, there may be a relationship between CNOT4 in LIHC cancer, and TAF1 may be associated with SARC cancer. However, only a few genes showed significant, and many cancer types found no significance. Based on the SNV analysis, while there were high mutation frequencies across several cancer types, the data suggests that protein autoubiquitination-associated genes might not be critical for most cancers. That being said, there may be some significance as it relates to UCEC, STAD, LIHC, and SARC cancers.

### Copy number variation analysis

**Fig. 2:**
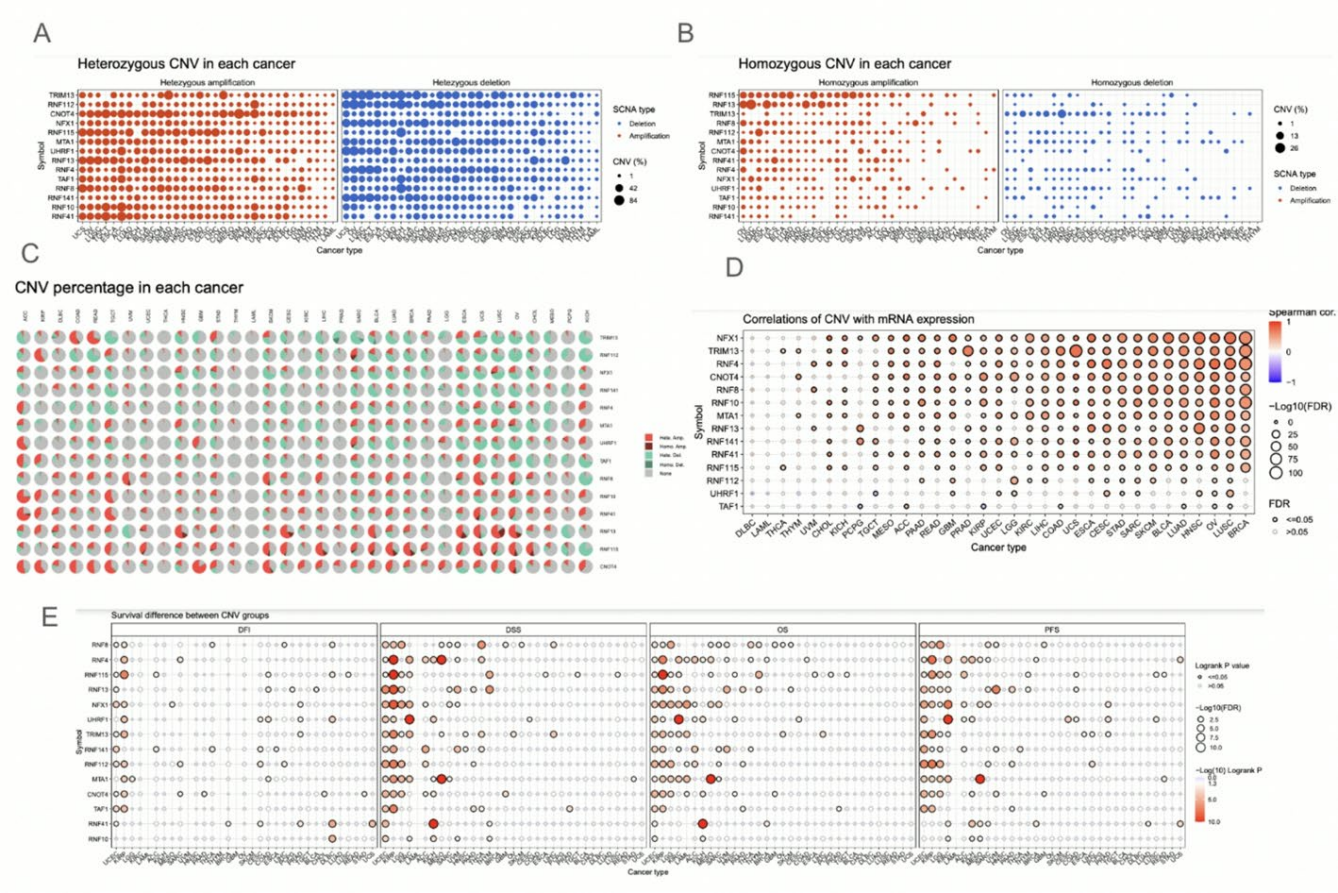
Copy-number alteration patterns and expression correlations. A: Heterozygous Copy Number Variations (CNV) of the genes across cancers. B: Homozygous CNV of the genes across cancers. C: Pie charts display CNV distribution across cancers. Homo Amp = homozygous amplification; Homo Del = homozygous deletion; Hete Amp = heterozygous amplification; Hete Del = heterozygous deletion. D: Correlations between CNV and mRNA expression. Spearman cor. is the correlation coefficient. E: Survival difference between CNV groups in protein autoubiquitination-associated genes

The copy number variation (CNV) of the protein autoubiquitination-associated genes was analyzed. The CNV profiles showed a lot of pattern variety in the genes across cancer types overall. The CNV plots and pie chart showed that there was a vast amount of both heterozygous and homozygous CNV across all cancer types. Even so, there were more heterozygous CNV than homozygous CNV overall (Fig. 2A-C). The CNV and mRNA expression correlation profile showed that CNV is positively correlated with most genes. BRCA, LUSC, and OV were most correlated with CNV with mRNA expression (Fig. 2D). The survival analysis revealed that many of the genes have an association with patient survival. In particular, KIRP, KIRC, and MESO were the cancer types that showed the most relation with survival times. For instance, RNF4, RNF115, RNF13, NFX1, TRIM13, RNF112, and TAF1 genes all were significant in KIRP as it relates to patient survival (Fig. 2E). Based on the CNV analysis, there may be a potential association between CNV of protein autoubiquitination genes and cancers.

### Methylation analysis

**Fig. 3:**
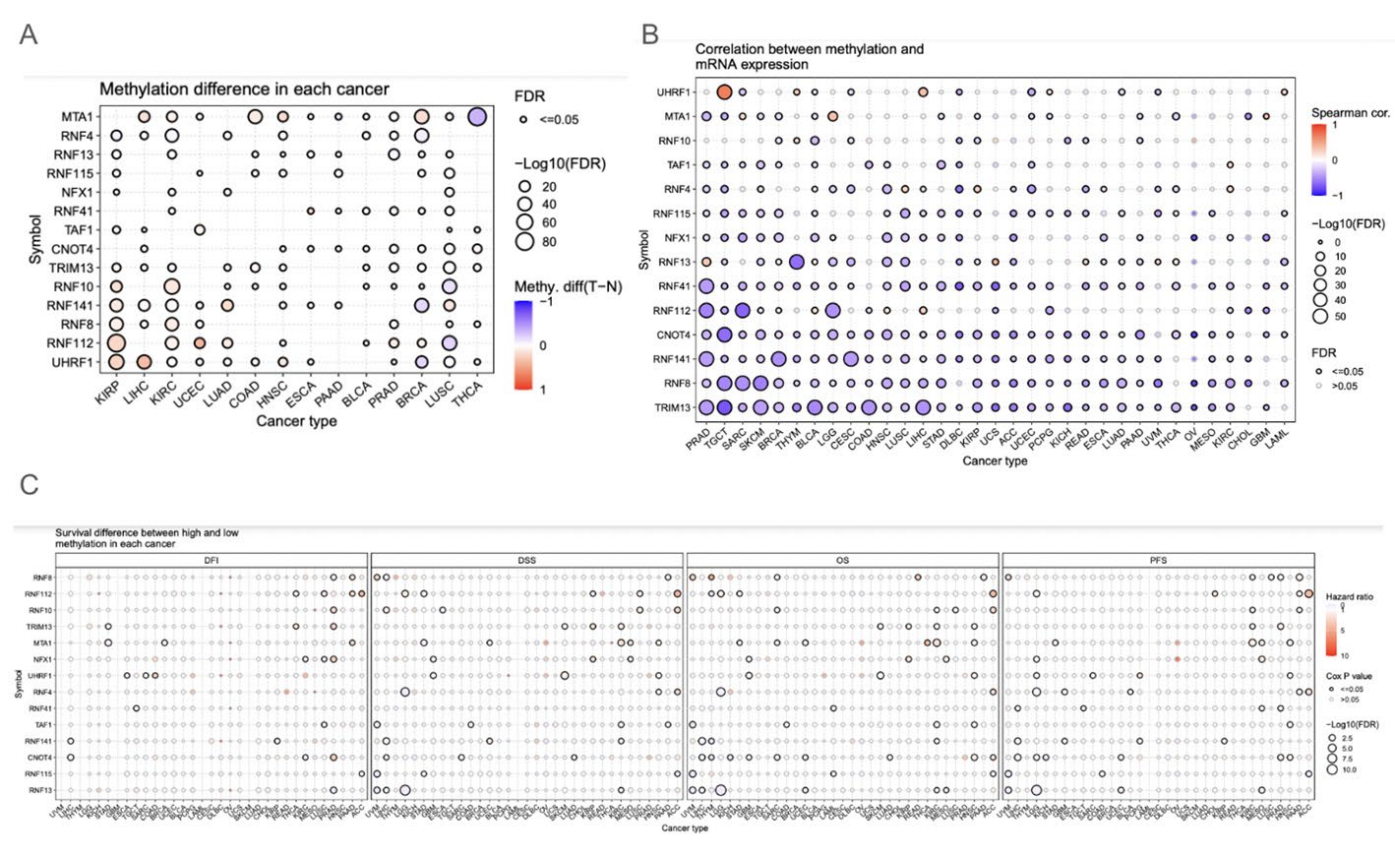
DNA methylation alterations and impact on gene expression and prognosis. A: Difference of methylation between cancer tumour and normal tissues. B: The correlation between methylation and mRNA expression. C: Survival difference between samples with high and low methylation of the genes

The methylation profile between tumor and normal cells revealed that there may be a difference between some cancer cells and normal tissue as it regards methylation levels. For instance, UHRF1 and RNF112 had higher methylation across some cancer types than normal cells (Fig. 3A). The correlation between methylation and mRNA expression showed that many genes displayed significance across a wide range of cancers, with the vast majority of these associations being negative correlations (Fig. 3B). The survival analysis revealed very few genes have significance between methylation and survival of patients (Fig. 3C). The methylation analysis revealed that there may be an association between hypermethylation and the down-regulation of genes in the protein autoubiquitination-associated genes.

### mRNA expression analysis

**Fig. 4:**
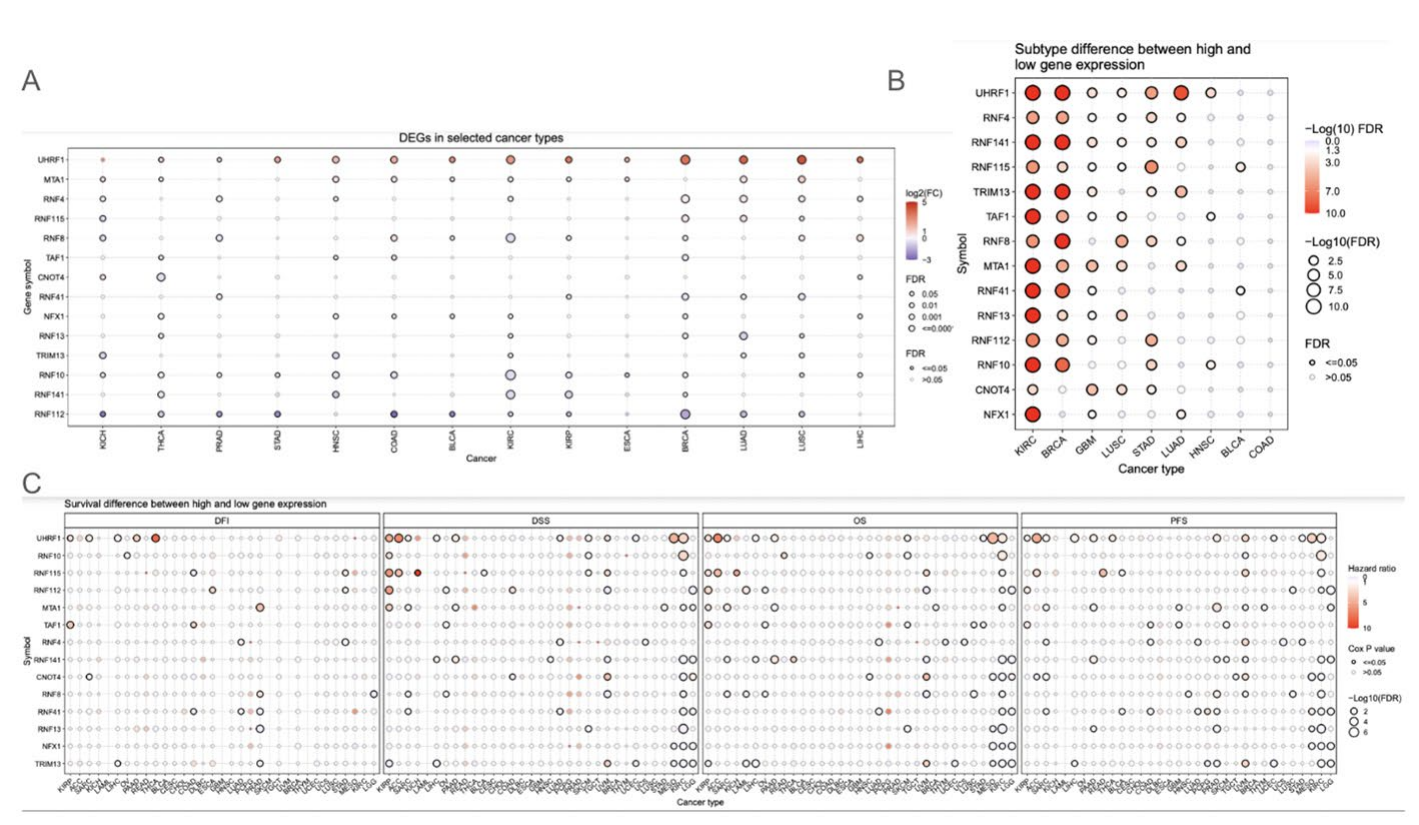
Transcriptomic dysregulation of autoubiquitination genes. A: Expression difference between normal and cancer tissues. B: Difference of expression between subtypes of cancers. C: Survival difference between high and low gene expression

The mRNA expression profiles of the gene set were analyzed. We examined the mRNA expression difference between cancer and non-cancer tissues of the protein autoubiquitination gene. The results indicated that there was some significance overall. For example, UHRF1 in LUSC and BRCA were upregulated, and a few other genes, like RNF10 and RNF8, were down-regulated in KIRC (Fig. 4A). The expression subtypes of these genes were also analyzed; the data revealed that KIRC and BRCA had the most significance in subtypes, but LUAD, LUSC, STAD, and GBM also showed some significance (Fig. 4B). The survival analysis revealed that some genes in KIRP, like UHRF1, have an impact on patient survival time. However, not much significance was revealed as only a few genes were significant (Fig. 4C). While there was significance as it relates to subtypes, the data revealed that the mRNA expression levels of these genes overall may not be critical for cancers.

### Crosstalk pathway analysis

**Fig. 5:**
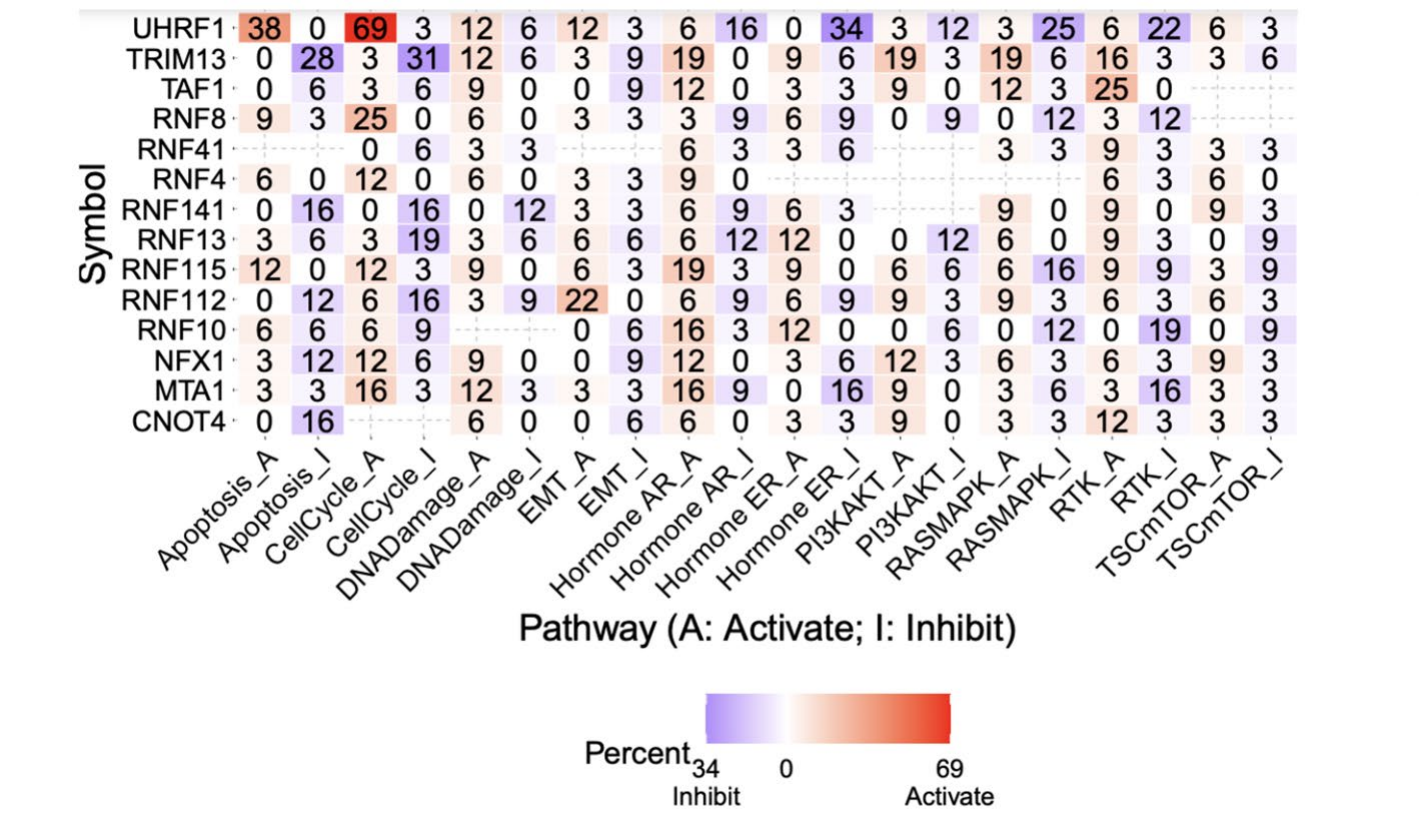
Integrated pathway analysis and clinical utility of autoubiquitination alterations. Expression and Pathway analysis and the predictive value of variations in the methylation of genes linked to protein autoubiquitination genes relating to patient survival

Potential cross-talk pathways between the protein autoubiquitination-associated gene set and other known cancer-related pathways were analyzed. High expression of many genes is associated with the activation of the epithelial-mesenchymal transition (EMT) pathway. 69% of cancer types analyzed associated high expression of UHRF1 with the activation of CellCycle_A. Other noteworthy genes included UHRF1 in Apoptosis_A, RNF8 in CellCycle_A, and TAF1 in RTK_A. Other genes were associated with the inhibition of the Hormone ER_I, like UHRF1 and MTA1 (Fig. 5). The pathway analysis revealed that there may be cross-talk between protein autoubiquitination-associated genes and other cancer pathways.

### Single cell analysis

**Fig. 6:**
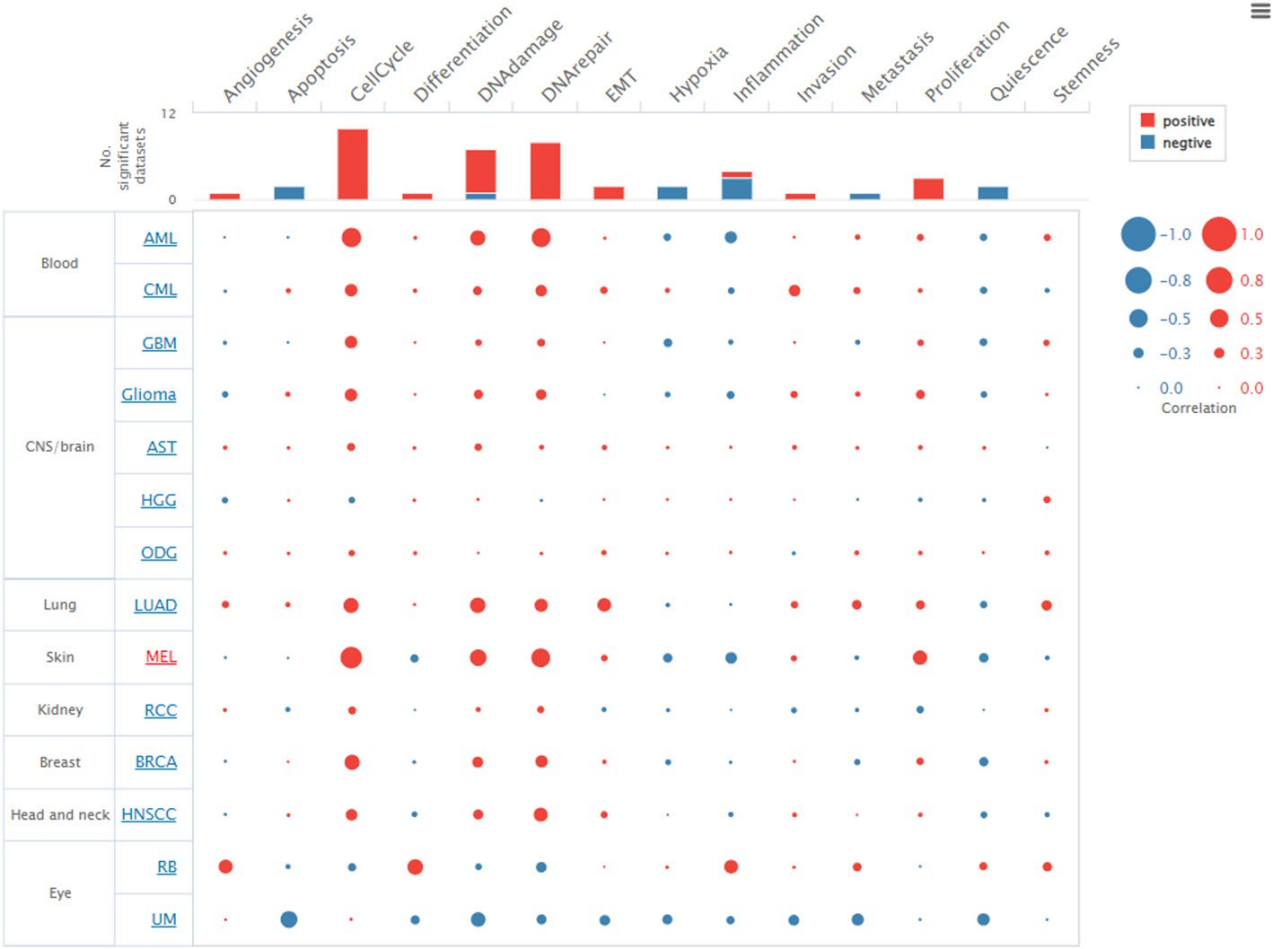
Correlation between cancer types and functional states from CancerSEA. The bubble plot displays the correlation between various cancer types (rows) and functional states (columns), derived from single-cell RNA-seq data using the CancerSEA database. Red bubbles indicate positive correlations and blue bubbles indicate negative correlations, with the size of each bubble representing the magnitude of the Spearman correlation coefficient. The intensity and direction of correlation are annotated in the legend (right), ranging from − 1.0 (strong negative) to 1.0 (strong positive). The top bar indicates the number of cancer types with significant correlations (*P* < 0.05) for each functional state

The association of genes like UHRF1 with multiple pathways indicates its central role in orchestrating complex cellular responses that may contribute to cancer cell survival and proliferation. Therefore, we conducted single cell analysis to explore its single cell pan-cancer profiles.To investigate the relationship between cancer functional states and tumor types, we analyzed single-cell transcriptomic data using the CancerSEA platform. As shown in Fig. 6, distinct cancer types exhibited varied correlations with 14 predefined functional states, including angiogenesis, apoptosis, cell cycle, differentiation, DNA damage, DNA repair, epithelial-mesenchymal transition (EMT), hypoxia, inflammation, invasion, metastasis, proliferation, quiescence, and stemness. Notably, melanoma (MEL) and glioblastoma (GBM) showed strong positive correlations with the cell cycle and proliferation states, suggesting high proliferative activity. In contrast, retinoblastoma (RB) demonstrated negative associations with multiple functional states, including inflammation and differentiation, while maintaining a positive correlation with apoptosis. Functional states such as cell cycle, DNA damage, and proliferation were among the most commonly correlated across cancer types, as indicated by the bar graph above the matrix. These observations support the heterogeneity of tumor biology and highlight cancer-type specific functional programs detectable at the single-cell level.

### Immune and drug sensitivity analysis

**Fig. 7:**
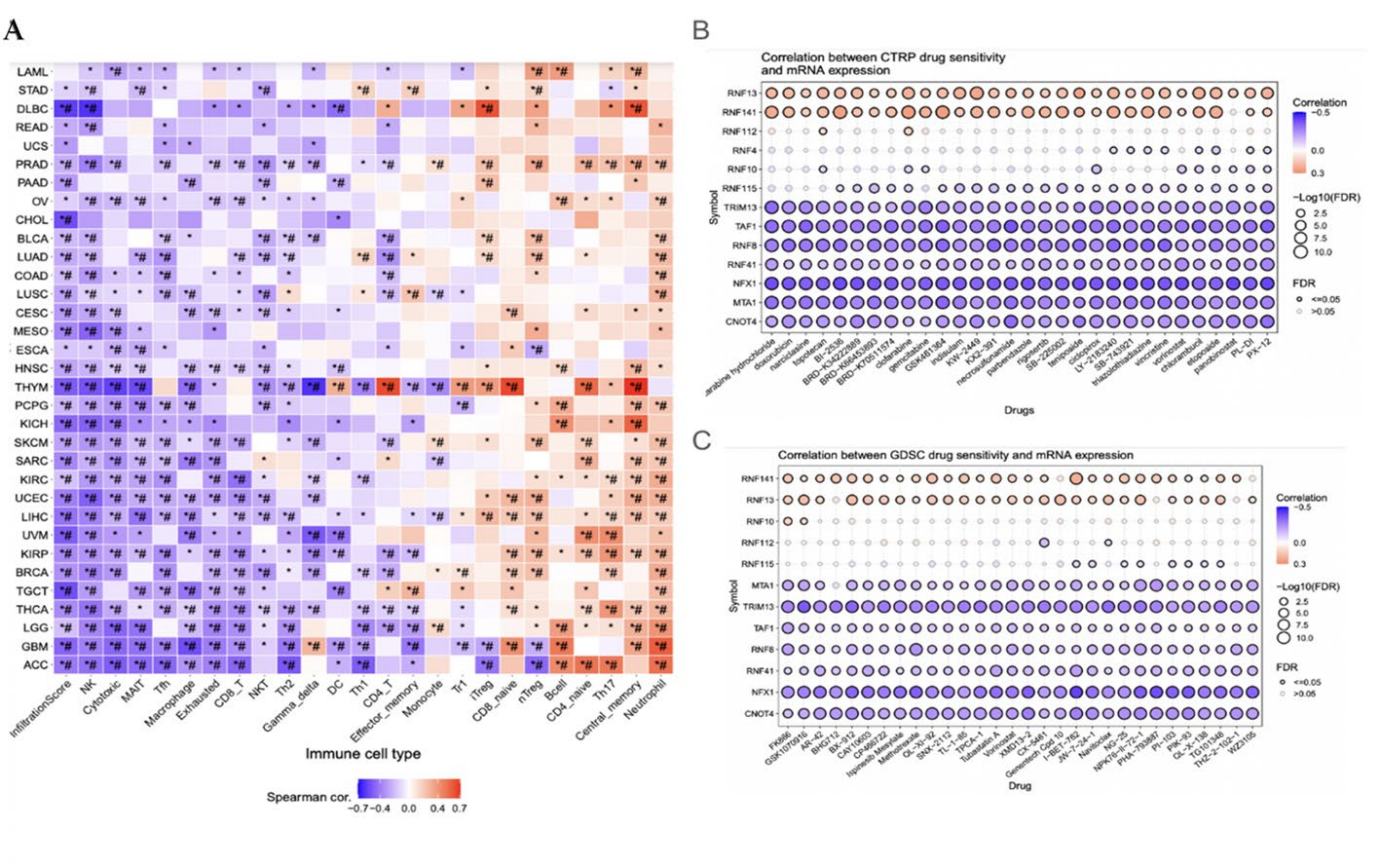
Immune infiltration and drug-sensitivity associations of autoubiquitination gene signature. A: A Correlation between immune cell infiltration levels (measured by ImmuCellAI) and GSVA scores of protein autoubiquitination-associated genes across cancer types; FDR < 0.05 and FDR < 0.01 denote statistical significance. B-C: Employing GDSC (B) and CTRP (C) data, showing the correlation between small molecule/drug sensitivity and the expression of the gene linked to protein autoubiquitination-associated genes in cancer cell lines. The association between gene expression and small molecule/drug sensitivity (area under the IC50 curve) was examined using Spearman correlation analysis

Lastly, this study explores the association between transcription factors of protein autoubiquitination genes and the immune cell mechanisms behind it. The GSVA score was used to do this analysis across multiple types of cancer. The data results showed that the GSVA score was somewhat associated with infiltration levels of immune cells, the main one included Central_memory with THYM (Fig. 7A). Additionally, the relationship between genes and the sensitivity of cell lines to the drug compounds was looked at through GDSC and CTRP databases. These results showed that most of these gene expressions are negatively correlated with predicted drug IC50s of multiple drugs, which means that a higher expression of these genes results in a higher sensitivity of these drugs, facilitating the cancer treatment (Fig. 7B). In conclusion, these major findings suggest that protein autoubiquitination genes could affect immune cell infiltration in cancers and potential drugs. The results show that there is a negative correlation between IC50 and low expression, meaning that the cancer is sensitive to the drug (Fig. 7C). This suggests that protein autoubiquitination genes are potential targets for immune therapy and chemotherapy for cancer.

## Discussion

Our comprehensive pan-cancer survey of autoubiquitination-associated transcription factors reveals both shared and cancer-type-restricted vulnerabilities. Notably, TAF1 mutations occur in over 20% of uterine corpus endometrial carcinoma (UCEC) samples, a frequency that correlates with decreased progression-free survival in this cohort, suggesting TAF1 as a candidate biomarker and potential therapeutic target in endometrial cancer. Similarly, NFX1 shows recurrent missense alterations in cutaneous melanoma (SKCM), while RNF10 is frequently mutated in colorectal adenocarcinoma (COAD), highlighting distinct mutation hotspots that may alter protein function in a context-dependent manner. The predominance of missense events across these genes implies that even subtle structural changes could disrupt key ubiquitin-mediated regulatory circuits and influence tumor behavior in specific cancer settings.

Differential expression and epigenetic profiling pinpoint UHRF1 and RNF115 as consistently overexpressed and hypermethylated, respectively, in breast (BRCA) and ovarian (OV) tumors, where their aberrant regulation has been linked to impaired DNA repair and unchecked proliferation. In BRCA and lung squamous cell carcinoma (LUSC), heterozygous amplifications of core autoubiquitination genes correlate strongly with increased mRNA levels, underscoring gene-dosage effects that may drive oncogenic programs. Pathway enrichment further connects these alterations to EMT and cell-cycle checkpoints, while immune deconvolution reveals that high GSVA scores of our autoubiquitination signature associate with reduced CD8⁺ T-cell infiltration in glioblastoma, pointing to an immunosuppressive microenvironment. Finally, inverse correlations between gene-set expression and IC₅₀ values for proteasome inhibitors in both GDSC and CTRP panels suggest that tumors with elevated autoubiquitination activity may be particularly susceptible to these agents, offering an actionable avenue for precision therapy.

In interpreting our drug-sensitivity correlations, it is important to emphasize that these data are strictly associative and do not establish causal relationships. To confirm whether altered expression of a given autoubiquitination transcription factor directly modulates drug response, future work should employ functional perturbation studies. For example, siRNA-mediated knockdown or CRISPR-Cas9 knockout of candidate genes [22] in cancer cell lines, followed by reassessment of IC₅₀ values for relevant compounds, would clarify causality. Complementary approaches, such as rescue experiments with ectopic gene re-expression, or use of inducible degradation systems, would further strengthen mechanistic insights.

While our integrative omics analysis provides a comprehensive landscape of autoubiquitination regulators and their association with drug responses in cancer, we recognize that the lack of direct experimental validation and the predominantly correlative nature of our findings limit the strength of mechanistic inferences. Moreover, although the breadth of data integration is a clear asset, the novelty of individual gene–drug associations awaits further substantiation. To enhance the impact and translational relevance of this work, future studies should prioritize targeted functional assays, such as siRNA/CRISPR perturbation coupled with phenotypic drug screens [22], to confirm the roles of top candidate ubiquitin ligases in modulating compound sensitivity. In addition, deep characterization of ubiquitination substrates through proteomic pulldown and mass-spectrometry will enrich the mechanistic context and may reveal previously unrecognized regulatory circuits. Finally, exploring synergistic effects between autoubiquitination modulators and standard-of-care therapies in in vivo cancer models will be critical for establishing both the novelty and therapeutic potential of these targets.

Pan-cancer omics analyses have become a staple in dissecting regulatory networks across tumor types, and while our focus on the autoubiquitination gene set represents a novel application, the overarching conceptual framework aligns with prior multi-dimensional studies [13, 23–26]. To delineate the unique, actionable contributions of the current work, we have now more clearly contrasted our findings with those from our earlier publications on related ubiquitin regulators. Specifically, unlike our prior focus on substrate-specific ligases, this study uncovers a coordinated module of autoubiquitination factors whose combined expression signature predicts sensitivity to deubiquitinase inhibitors and proteasome modulators. This integrative signature not only refines patient stratification, by identifying a subset of high-risk tumors with co-elevated E3 ligases, but also suggests immediate translational avenues, such as repurposing FDA-approved proteasome inhibitors in patients whose tumor profiles match the ‘autoubiquitination-high’ cluster. Moreover, the interdisciplinary application of generative adversarial networks (GANs) to gene-expression analysis offers a powerful means to synthetically generate RNA-seq profiles, filling critical gaps in transitional phenotypic states and accelerating biomarker discovery by reducing both computational burden and manual curation time [27].

In conclusion, this study underscores the multifaceted roles of protein autoubiquitination-associated transcription factors in cancer, highlighting their involvement in genetic, epigenetic, and expression-level alterations. The potential of these factors to serve as biomarkers for cancer diagnosis, prognosis, and therapy, especially in precision medicine, is significant. Future research should aim to elucidate the specific mechanisms by which these factors influence cancer pathways and to explore the therapeutic potential of targeting these mechanisms in diverse cancer contexts.

### Limitations

This study is constrained by its reliance on heterogeneous, publicly available datasets without accompanying in vitro or in vivo functional validation, which limits causal inference. Platform-specific biases, such as RNA-seq batch effects, differences in array platforms, and data processing pipelines, may introduce systematic variability and confound cross-cohort comparisons [28, 29]. Our single-cell analyses, while informative, cover only a subset of tumor types and may not capture rare cell populations or dynamic state transitions. Correlations between gene expression and drug sensitivity are associative and do not establish mechanistic links. Finally, the absence of longitudinal clinical outcome data and detailed treatment histories restricts our ability to assess prognostic value over time. Future studies should incorporate standardized preprocessing to mitigate batch and platform effects, expand single-cell profiling across diverse cancers, and include longitudinal cohorts with experimental perturbations to validate causal roles and clinical utility.

## Data Availability

The source of the raw data was provided in the paper and the raw analysis data of this study are provided by the corresponding author with a reasonable request.
